# Are SNPs Linked to Somatic Cell Score Suitable Markers for the Susceptibility to Specific Mastitis Pathogens in Holstein Cows?

**DOI:** 10.1111/jbg.12904

**Published:** 2024-11-01

**Authors:** U. Müller, E. M. Strucken, J. Gao, S. Rahmatalla, P. Korkuć, M. Reissmann, G. A. Brockmann

**Affiliations:** ^1^ Breeding Biology and Molecular Genetics, Department for Crop and Animal Sciences Humboldt‐Universität Zu Berlin Berlin Germany

## Abstract

Mastitis in cattle is often caused by microorganism infections in the udder. The three most common pathogens are esculin‐positive streptococci (*SC+*), coagulase‐negative staphylococci (*CNS*), and *Escherichia coli* (*E. coli*). In a previous study, 10 SNPs were associated with somatic cell score and mastitis in diverse Holstein populations. We tested these SNPs for their effects on individual pathogen presence. Milk and pathogen samples of 3076 Holstein cows were collected from four farms. Samples were excluded if multiple pathogens were present at the same time. Records of the same pathogen within 14 days of each other were counted as one infection. This resulted in 1129 pathogen‐positive samples. Cases and controls were in ratios of 20:80 for *SC+*, 8:92 for *CNS*, and 11:89 for *E. coli*. The lasso, backward, and forward methods were used to narrow down SNPs associated with pathogen presence. The suitability of the SNPs to separate the samples into cases or controls for each pathogen was indicated using ROC curves. The Cochran‐Armitage (CAT) and the Jonckheere‐Terpstra (JTT) tests evaluated the influence of the SNPs on pathogen presence. Finally, a generalised linear mixed model (GLMM) including fixed environmental effects and a random sire effect was fitted to the binary trait of pathogen presence to test for association. In total, six out of the 10 investigated SNPs showed associations with pathogen presence based on the forward method: Two SNPs each for *SC+* (rs41588957, rs41257403) and *CNS* (rs109934030, rs109441194), and three for *E. coli* (rs109934030, rs41634110, rs41636878). The CAT and GTT tests linked four SNPs (rs41588957, rs41634110, rs109441194, rs41636878) to pathogen presence, two of which were confirmed with the GLMM (rs41634110, rs109441194), with effects on *CNS* and *E. coli*. The SNPs linked to *CNS* and those linked to *E. coli* explained 13.2% and 13.8% of the variance, compared to 19% and 18.4%, respectively, of the full model with all 10 SNPs. Half of the SNP genotypes previously linked to lower SCS also decreased the probability for pathogen presence and might therefore be targets not just for lower SCS but for a better pathogen resistance.

**Trial Registration:** Not applicable, no new data were collected for this study.

## 1 Introduction

Mastitis in cows is an inflammation of the mammary glands with multifactorial causes (De Vliegher, Ohnstad, and Piepers 2018). Population management has the largest impact on udder health, but even under the best production environment, cows develop mastitis (Abebe et al. 2016; Pyörälä et al. 2008; Stein and Katz 2017; Valde et al. 2007). Olde Riekerink et al. (2008) analysed 106 herds from different provinces in Canada and found on average 23% of clinical mastitis cases. The average incidence rate per year was between 0.08 and 0.32.

The application of vaccines to prevent udder infections is restricted due to limited understanding of their effectiveness, and antibiotics resistance is increasing, limiting their application in the field (Rainard et al. 2021). Animal breeding can contribute to decreasing mastitis incidences by selecting animals that show resistance to mastitis infections. In genomic selection, animals with a favourable genomic makeup for desirable traits are selected. Several investigations across various Holstein populations have explored genomic regions linked to somatic cell score (SCS) or clinical mastitis, pinpointing these areas to chromosomes 1, 5, 6, 8, 13, 16, 18, 19, and 20 (Abdel‐Shafy, Bortfeldt and Tetens 2014, Abdel‐Shafy, Bortfeldt and Reissmann 2014, 2018). However, marker associations with mastitis, somatic cell count, or its log‐transformed somatic cell score (SCS) have rarely been replicated between studies (Narayana et al. 2023), and a negative correlation between reduced somatic cell counts and increased milk production makes a simultaneous selection for both traits difficult (Hagnestam‐Nielsen et al. 2009).

For an effective selection strategy, especially against frequent and aggressive mastitis pathogens, the causes for the infection must be comprehensively recorded. *Streptococcus uberis*, *E. coli*, coagulase‐negative staphylococci, *Staphylococcus aureus* and *Streptococcus agalactiae* have been repeatedly documented as the most prevalent pathogens in modern dairy herds (Heikkilä et al. 2018; Krömker and Leimbach 2017; Bianchi et al. 2019; Duse, Persson‐Waller, and Pedersen 2021; de Haas, Barkema, and Veerkamp 2002; Cobirka, Tancin, and Slama 2020). While different mastitis pathogens have different effects on somatic cell counts in milk (de Haas, Barkema, and Veerkamp 2002), the susceptibility and response of an individual to become ill or to fight the pathogen is variable and may differ between pathogens.

The question is whether genetic markers identified for SCS could, therefore, also be used to predict resistance against specific pathogens. In the present study, we investigated 10 SNP markers previously associated with SCS (Abdel‐Shafy et al. 2014; Elzaki et al. 2022), five of which were also found to be associated with clinical mastitis (Abdel‐Shafy et al. 2018).

## 2 Material and Methods

### 2.1 Data

Data were collected from 3076 German Holstein Friesian cows kept under conventional production environments on four farms. The farms had between 357 and 1724 cows with 565 to 2794 lactation records. The first to third lactations were analysed separately, and the fourth and later lactations were grouped as one. Approximately half of the animals had only one lactation recorded in the dataset. Cows in the first lactation (*n* = 816) had a lower milk yield after parturition (29.3 ± 6.68 kg) compared to cows in later lactations (40.93 ± 9.07 kg), however, total milk yield was almost equal between primiparous and multiparous cows (first lactation: 10,285.22 ± 3359 kg; later lactations: 10,166.94 ± 4495.23 kg).

Milk samples for bacterial determination were collected at the beginning of every lactation and in cases of increased somatic cell counts or externally visible inflammation of the udder. Sampling was conducted between January 2016 and October 2019 (Table 1).

**TABLE 1:** Farm data of four dairy herds for milk yield and somatic cell counts (2016–2019).

Farms	Number of cows	Average number of lactations per cow	Average milk yield kg over 305 days	Average SCS	Median SCC^a^ (SC/mL)
1	1724	2.3	9963	2.6	61,000
2	357	2.3	9740	2.6	61,000
3	586	2.6	11,398	2.8	67,000
4	409	2.5	10,808	2.5	58,000

Abbreviation: SCS = (log_2_ (somatic cell count/100.000)) + 3.

^a^ Rounded to full thousand; median is presented due to the skewness of SCC data.

The classification of mastitis pathogens in the milk samples was carried out by an accredited laboratory. The pathogen spectrum tested by the laboratory included the five most common pathogen groups, namely esculin‐positive streptococci (*SC*+), esculin‐negative streptococci, *E. coli*, coagulase‐negative staphylococci (CNS), and *S. aureus*. Samples were taken on all farms from cows exhibiting clinical and sub‐clinical mastitis symptoms. The status of clinical or sub‐clinical mastitis was not recorded and therefore could not be distinguished in the available data. A large percentage of the samples were excluded due to other bacterial contaminations with no additional specifications regarding the pathogen.

All farm data, such as milk performance, animal health, and inseminations were entered by the farms into the herd management system HERDE and HERDEplus (DSP Agrosoft Paretz). Coding of disease entries followed the recording system recommended by the central animal health registry (Zentraler Tiergesundheitsschlüssel ZTGS RIND) which was established in 2008 as the recording standard in Germany.

### 2.2 Pathogens and Data Management

A case–control design was used to analyse the data, requiring that all mastitis cases can be unequivocally distinguished from the control group, but is in all other factors as similar as possible. The control group included animals without a mastitis or pathogen record or other symptoms of any disease in any lactation. The case group included all sick animals exhibiting a specific mastitis pathogen in at least one milk sample throughout a lactation. Reports of the same pathogen within 14 days were treated as one incident. Samples were excluded if other bacterial contaminations prohibited an exact proof of a mastitis pathogen, or if the mastitis pathogen was detected after lactation day 305.

After cleaning the data, three pathogen classes (esculin‐positive streptococci, coagulase‐negative staphylococci, and *E. coli*) with sufficient animal records remained (Table 2; Table S1).

**TABLE 2:** Number of lactation records as well as the number of cases and controls for every pathogen recorded for all milk samples across four herds after quality control.

	*SC+*	*CNS*	*Escherichia coli*	Total
Number of lactations	3783	1841	2072	7696
Number of controls	3032	1696	1839	6567
Number of cases	751	145	233	1129

### 2.3 Genotypes

Ten SNPs were selected that had been previously linked to mastitis or SCS in Holstein cows (Abdel‐Shafy et al. 2014). There was no overlap between the animals of this study and the animals analysed by Abdel‐Shafy et al. (2014). The 10 selected SNPs are located on chromosomes 5, 6, 13, 18, 19, and the X‐chromosome. Chromosomes 6 and 19 contained two SNPs each, and chromosome 13 had three SNPs of interest (Table 3). Genotyping was performed using allele‐specific KASP assays as published before (Abdel‐Shafy et al. 2014; Kreuzer, Reissmann, and Brockmann 2013). Genomic positions refer to the Bovine ARS‐UCD1.2 genome assembly.

**TABLE 3:** Population parameters of the 10 SNPs under investigation.

SNP label	SNP rs	Chr	Position bp	Major allele	Minor allele	MAF	HWE (*α* = 0.05)			
							Farm1	Farm2	Farm3	Farm4
SNP1	rs41257360	5	97,477,421	A	G	0.09	yes	yes	yes	yes
SNP2	rs41588957	6	83,803,915	T	C	0.12	yes	yes	yes	yes
SNP3	rs110707460	6	86,337,334	A	G	0.05	yes	no	yes	yes
SNP4	rs109934030	13	77,914,930	C	T	0.01	yes	yes	yes	yes
SNP5	rs41634110	13	79,002,832	A	G	0.13	yes	no	yes	yes
SNP6	rs109441194	13	79,365,467	T	C	0.23	yes	yes	yes	yes
SNP7	rs29020544	18	43,157,279	T	G	0.20	yes	yes	yes	yes
SNP8	rs41257403	19	50,027,458	G	A	0.01	yes	yes	yes	yes
SNP9	rs41636878	19	51,815,015	C	T	0.05	no	yes	yes	yes
SNP10	rs41629005	X	30,341,984	T	C	0.02	yes	yes	yes	yes

Abbreviations: HWE, Hardy–Weinberg equilibrium; MAF, minor allele frequency.

### 2.4 Statistical Analyses

All analyses were carried out within SAS 9.4. (SAS‐Institute 2002–2012). The pathogens were recorded separately and cows with pathogen verification in the case group were coded as CASE = 1 and healthy cows in the control group as CONTROL = 0.

In the first step, the 10 investigated SNPs were tested for their suitability to separate the animals into the case or control group. Three separate methods were used, namely the lasso, a backward, and a forward selection which sequentially added or removed SNPs from the model. The best SNP combination for each pathogen was determined based on the Akaike information criterion (AIC). The AIC estimates the prediction error of a model, providing the best balance between model fit and number of estimated parameters. The smaller the value the better the model fit.

A ROC curve (Receiver Operating Characteristic) was used to assess how well cases and controls could be distinguished based on the SNP genotypes. Correctly assigned cases (sensitivity) are compared to wrongly assigned cases (1‐specificity) which determines the area under the curve (AUC), also known as the concordance index C. The AUC can take values between 0.5 and 1 to describe the discrimination ability of a predictive model, the larger the value the better the fit of the model.

In a second step, we tested each SNP separately regarding whether the genotypes were differently distributed between the case and control groups. The Cochran‐Armitage Test (CAT) and the Jonckheere‐Terpstra test (JTT) are often used in genetic association analyses. The JTT showed larger power in cases of non‐additive allele effects and small minor allele frequencies (Manning et al. 2023). *p*‐Values < 0.05 were interpreted as significant effects, and values < 0.1 were reported as tendencies.

In a third step, a generalised linear mixed model (GLMM) was used to also include environmental effects. The selected SNP genotypes in their combination as determined in step 1 were included with environmental effects into a binary logit model. The entries for CASE/CONTROL (1/0) were log‐transformed as follows:

$$\log\left[\frac{\pi_{i}}{1-\pi_{i}}\right]=\eta_{i}$$

where *π*_*i*_ is the probability that a pathogen was detected. Thus, $\eta_{i}$ is the logarithm of the probability of a pathogen detection over the probability that no pathogen was present.

The complete logit model can be written as:

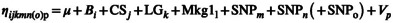

where *μ* is the mean across all farms, *B*_*i*_ is the fixed effects of the farm *i*, CS_*j*_ is the fixed effect of the calving season *j* (*j* = 7; April to September and October to March per year), and LG_*k*_ is the fixed effect of the lactation group *k* (first to third lactation separately, fourth and later lactations grouped as one). Pathogens for *E. coli* and *CNS* occurred less often than for *SC+*, therefore, only 2 lactation groups were formed for *E. coli* and *CNS*, and 4 groups for *SC+*. Mkg1_l_ is the fixed effect of milk yield at the beginning of the lactation, SNP_(*m, n, o*)_ are the fixed effects of the SNP genotypes in the combination as determined in step 1 for each pathogen, and *V*_*p*_ is the random effects of the sire p (536 sires).

The distribution of the dependent variable (*Y*_*i*_) and the random effect of the father (*V*_*p*_) can be described as follows:

$$Y_{i}|V_{p}\sim \text{binomial}\left(n_{i},\pi_{i}\right)$$

$$V_{p}\sim \text{iidN}\left({0,\sigma }_{c}^{2}\right)$$

No father‐son pairs were recorded and additive relationships between sires were ignored. All least square means (LSMEANS) for the genotypes were back‐transformed with a logistic function. Bonferroni correction was applied to the *p*‐values since 2 or 3 SNPs (*n*) were tested simultaneously. *p*‐Values < 0.05/*n* were interpreted as significant effects, and values < 0.1/*n* were reported as tendencies.

## 3 Results

Esculin‐positive streptococci (*SC+*) were the most prevalent mastitis pathogen on all four farms and occurred in 53.4% of all samples (range between herds 30.1%–67.9%), followed by *E. coli* with 15.1% (8.4%–18.8%), and coagulase‐negative staphylococci (*CNS*) with 12.9% (9.2%–35.4%). *S. aureus* 9.6% (1.4%–25%) and esculin‐negative streptococci 9.0% (8.0%–15.8%) had an average incidence rate below 10% and were not analysed further.

SNP3 and 5 were not in Hardy–Weinberg equilibrium (HWE) for herd 2, and SNP9 was not in HWE in herd 1 (Table 3).

The lasso method did not detect any significant SNP effects on pathogen presence. The backward and forward selection methods, however, identified significant combinations of SNPs that could influence the occurrence of pathogens. The combination of SNPs 2 and 8 was a significant descriptor for the case and control grouping for *SC+*, and the combination of SNPs 4, 5, and 9 for *E. coli*. For *CNS*, only SNP6 was chosen in the forward selection and the combination of SNPs 4 and 6 in the backward selection method (Table 4).

**TABLE 4:** SNPs selected in different models and model‐dependent AUC values.

	Selection method		Logistic model		
Pathogen	Backward	Forward	Model	AUC	∆AUC
			NULL	0.500	
*SC+*	SNP2 & 8	SNP2 & 8	SNP2 & 8 FULL	0.533 0.555	+6.6% +11.1%
*CNS*	SNP6	SNP4 & 6	SNP6 SNP4 & 6 FULL	0.547 0.566 0.595	+9.4% +13.2% +19.0%
*Escherichia coli*	SNP4, 5, & 9	SNP4, 5, & 9	SNP4, 5, & 9 FULL	0.569 0.592	+13.8% +18.4%

Abbreviation: AUC, area under the curve.

The AUC was calculated for the different combinations of selected SNPs as well as for a NULL model (no SNPs) and a FULL model (all 10 SNPs). A value of 0.5 (NULL model) indicates that cases and controls cannot be separated based on the provided genotypes, and an AUC of 1 would indicate that the model can distinguish between cases and controls perfectly.

All AUC values were between 0.5 and 0.6 and neither the selected SNPs nor the FULL model allowed for an accurate separation of cases and controls. However, the selected SNPs increased the accuracy of the estimate. Compared to the FULL model, the selected SNPs explained more than 50% of the AUC (Table 4).

In the second step, we tested whether the investigated SNPs are suitable as genetic markers to explain the presence of pathogens in the milk using the Cochran‐Armitage and the JTT. Most notably, SNP5 and SNP9 were significantly associated with the presence of *E. coli* in both tests. *SC+* was significantly impacted by SNP2 based on the JTT and showed a trend for association based on the Cochran‐Armitage test, and *CNS* was significantly associated with SNP6 based on the Cochran‐Armitage test and showed a tendency for association based on the JTT (Table 5).

**TABLE 5:** *p*‐Values for selected SNPs from the Cochran‐Armitage Test (CAT) and the Jonckheere‐Terpstra Tests (JTT).

Pathogen	Test	SNP2	SNP4	SNP5	SNP6	SNP8	SNP9
*SC+*	CAT	0.074	—	—	—	0.308	—
	JTT	**0.046**	—	—	—	0.188	—
*CNS*	CAT	—	0.226	—	**0.035**	—	—
	JTT	—	0.251	—	0.070	—	—
*Escherichia coli*	CAT	—	0.761	**0.005**	—	—	**0.016**
	JTT	—	0.498	**0.007**	—	—	**0.012**

*Note*: The bolded values are significance values < 0.05.

The third step estimated SNP effects on the presence of mastitis pathogens simultaneously and accounted for environmental effects. The environmental effects reduced the genetic impact of the selected SNPs. For *E. coli*, SNP5 remained significant and SNP9 showed a tendency (*p* < 0.1) for an effect, while SNP6 remained significant for *CNS* (*p* = 0.038). *SC+* was significantly influenced by all four environmental factors (*p* < 0.0001), *CNS* only by farm and calving season (*p* < 0.01), and the presence of *E. coli* was impacted by early milk yield, calving season, and lactation group (*p* < 0.003), but not by the farm (Table 6).

**TABLE 6:** Overview of *p*‐values for the selected SNPs and fixed effects from the GLMM analysis for three different mastitis pathogens.

	*SC+*	*CNS*	*Escherichia coli*
SNP2	0.138	—	—
SNP4	—	0.194	0.138
SNP5	—	—	**0.049**
SNP6	—	**0.038**	—
SNP8	0.146	—	—
SNP9	—	—	0.059
Farm	< 0.001	0.01	0.701
Calving season	< 0.001	< 0.001	0.003
Lactation group	< 0.001	0.366	0.002
First milk yield	< 0.001	0.501	0.002

*Note*: The bolded values are significance values < 0.05.

**FIGURE 1:**
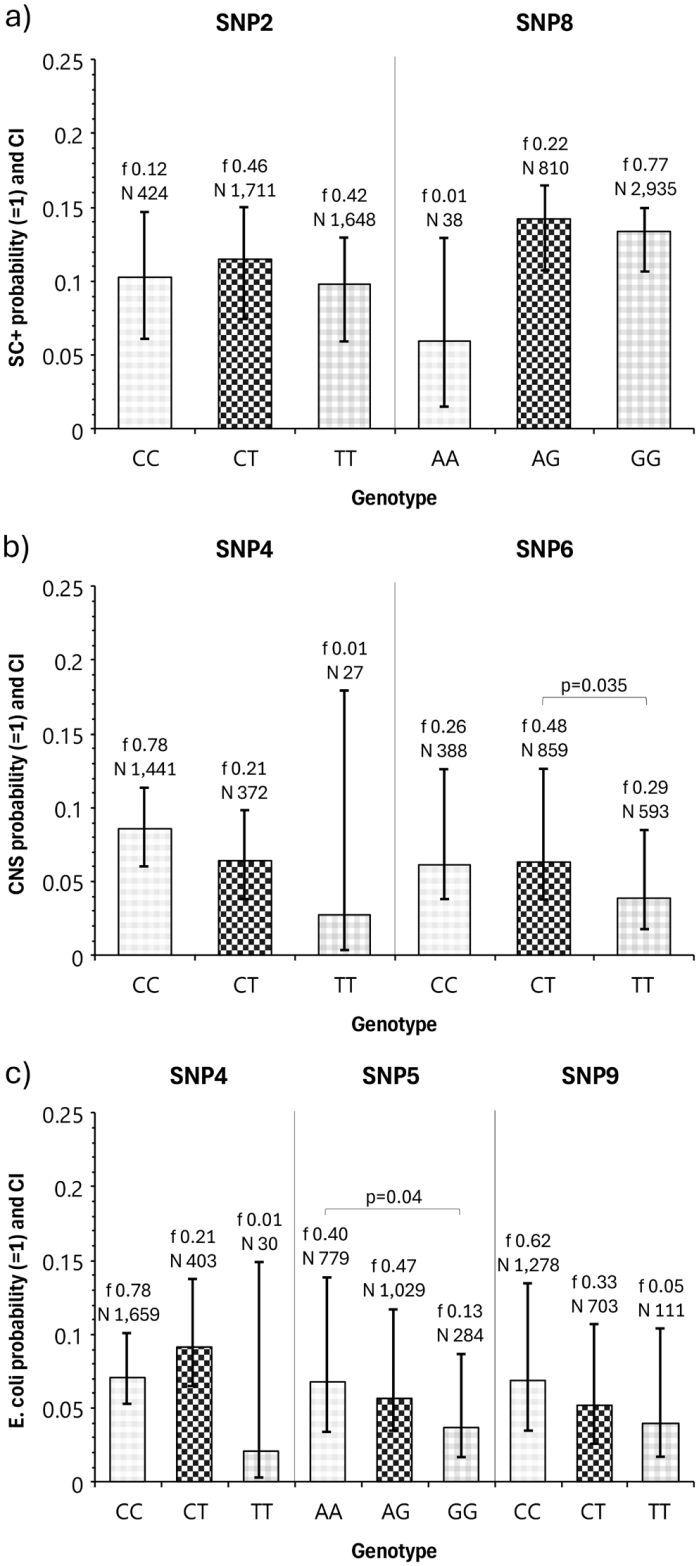
Probability (with 95% confidence intervals CI) for the occurrence of (a) *SC+*, (b) *CNS*, and (c) *E. coli* in milk samples, depending on selected SNP genotypes. Values above the columns represent the genotype frequency *f* and the number of lactations *N* that were tested. The Bonferroni‐adjusted *p*‐values indicate significant differences between genotypes.

For all but two SNPs (SNP2 and 6), the minor allele was associated with the genotype that indicated a lower probability for pathogen presence. For *SC+*, no significant difference between the genotypes of SNP2 and SNP8 was found regarding the probability of the presence of the pathogen (Figure 1a). For *CNS*, the heterozygous genotype of SNP6 had a significantly higher probability for pathogen presence compared to the homozygous genotype of the major allele (*p* < 0.035) (Figure 1b). The homozygous genotype of the minor allele had a higher probability for pathogen occurrence compared to the major allele genotype, but the difference was not significantly different to the other genotypes. For *E. coli*, the group of the homozygous genotype with the minor allele (GG) of SNP5 had significantly the lowest probability of pathogen occurrence (*p* < 0.04; Figure 1c).

## 4 Discussion

The German National Information System For Animal Husbandry (VIT) has estimated breeding values for somatic cell count (heritability 0.16–0.17) since 1996 and expanded its recording to mastitis resistance (EFit) in 2019. For Holstein Friesian dairy cattle, mastitis resistance is estimated to have a low heritability of 0.08 (VIT Verden; Documentation of Breeding Value Estimation, 2024). However, mastitis is caused by various pathogens and the immune reaction could be controlled by different pathogen‐specific loci. We examined 10 loci in the genome previously associated with mastitis and SCS (Abdel‐Shafy et al. 2014), to determine their contribution regarding the genetic differentiation in mastitis susceptibility depending on the detected pathogen type.

The occurrence of the pathogen *SC+* was mostly impacted by SNP2 and SNP8. In the Holstein population investigated by Abdel‐Shafy et al. (2014), the homozygous genotype of the minor allele of SNP2 was associated with an increased somatic cell count. However, we did not find that the SNP impacted the occurrence of *SC+* pathogens in the milk if environmental factors were considered. Similarly, for SNP8 no significant differences between genotypes were found, however, the minor allele genotype AA had the lowest probability that the pathogen was present in the milk sample. Abdel‐Shafy et al. (2014) also found that this genotype of SNP8 was associated with the lowest somatic cell score.

SNP8 is located on BTA19 near the *forkhead box K2* (*FOXK2*) gene. This gene was reported to be involved in the IL‐2 mRNA synthesis in active T‐lymphocytes in pigs (Xu et al. 2023). Further, lactating cows with a clinical mastitis showed a significant increase in IL‐2 compared to healthy animals. Differences in subclinical mastitis cases were less pronounced (Shaheen et al. 2020). Our findings suggest that infection with *SC+* could affect the immune system through the activation of IL‐2 expression.

The presence of *CNS* was impacted most by SNP4 and SNP6. Both markers are located on BTA 13 within 1.5 Mbp of each other. For SNP4, the homozygous minor allele genotype TT showed the lowest probability for pathogen presence, albeit with a large confidence interval and non‐significant. Nonetheless, this contrasts with Abdel‐Shafy et al. (2014), who reported that the TT genotype was associated with an increase in somatic cell count. It is important to note that both our study and the study by Abdel‐Shafy et al. (2014) only had a small number of records with the rare TT genotype, which most likely contributed to the deviating results and the large confidence interval.

SNP6 showed a significant impact on *CNS* presence in the Cochran‐Armitage test and the GLMM analysis. This is the only SNP where the homozygous major allele genotype was advantageous and showed a significantly lower probability for mastitis compared to the heterozygous genotype. The probability for a *CNS*‐induced mastitis was similar for the homozygous minor allele genotype CC and the heterozygous genotype; however, differences were not significant most likely due to the low number of lactation records for the minor allele genotype. Our study aligns with the results of the bull population of Abdel‐Shafy et al. (2014) where the TT‐genotype was associated with a favourable somatic cell count.

SNP6 is located near the *nuclear factor of activated T cell 2* (*NFATC2*) gene. This gene encodes a protein present in activated T cells and is involved in immune reactions during the induction of interleukins (Macian 2005).

Despite the presented results, we cannot unequivocally confirm from the current study that SNP6 is sufficiently informative to act as a marker for somatic cell count or the presence of *CNS* pathogens. The correspondence between our results and Abdel‐Shafy et al. (2014) for bulls indicates nonetheless that selection against somatic cell score based on SNP6 genotypes could reduce the probability for a *CNS*‐mastitis.

Two out of the three SNPs tested for *E. coli* remained significant or at least with a tendency in multiple tests. The homozygous minor allele genotypes of all three SNPs had the lowest probability for an *E. coli* infection which is in concordance with Abdel‐Shafy et al. (2014). SNP4 and SNP5 are both located on chromosome 13 within a region of 1 Mbp. SNP4 was not significant in any tests, however, SNP5 was significantly associated with mastitis in all tests, even the GLMM.

Abdel‐Shafy et al. (2014) reported for SNP5 slightly reduced somatic cell scores for the minor allele genotype GG in cows compared to the other genotypes. We confirm that cows with the GG genotype had the lowest probability for an *E. coli* mastitis. Therefore, selection against somatic cell score based on SNP5 genotypes could also reduce the occurrence of *E. coli* mastitis.

SNP5 is in an intron of gene ENSBTAG00000049099 which transcribes lncRNA. Such RNAs play a key role in disease expression by regulating the transcription of neighbouring genes involved in the function of nuclear condensates or post‐transcriptional regulation of protein synthesis (Statello et al. 2021). Nothing has been reported so far as to the involvement of ENSBTAG00000049099 in infectious diseases in humans or animals.

SNP9 is located on chromosome 19 and was only significant in the Cochran‐Armitage and the JTT. The SNP is located in an intron region of the *regulatory associated protein of MTOR complex 1* (*RPTOR*) gene. This gene encodes a protein involved in cell growth including the development and function of connective tissue (Lei 2019). In cattle, the gene is also involved in the expression of milk cholesterol content (Do et al. 2018). Further, the involvement of the gene in the development of natural killer cells is of interest in connection to mastitis resistance (Yang et al. 2018; Foerster et al. 2022).

Logit models have a lower power compared to models using a continuous dependent variable. Repeated continuous variables on the same individual, as it is the case with somatic cell score in milk for example, increase the accuracy of the estimates.

The pathogens causing mastitis were not known in the work of Abdel‐Shafy et al. (2014). Differences in the occurrence of pathogens between the herds examined in this study and in the study of Abdel‐Shafy et al. (2014) could have impacted the somatic cell score and caused the differences in results.

## 5 Conclusions

The selected SNPs had a low impact on the reliabilities with which an animal could be partitioned into the case or control group based on their genotype alone. Strongly significant effects were not found, but genotypes that decreased the probability for the presence of a pathogen were also linked to a lower SCS.

Although the effects of the utilised markers are small on their own, they are positive and impactful as a sum and explained more than half of the effect size compared to the ten originally selected SNPs together, based on the AUC values.

Targeted selection for specific pathogen resistance, compared to using a secondary trait such as somatic cell counts, could increase the power of selection against mastitis by focussing on the cause (pathogen resistance) rather than the effect (increased SCC). Additionally, a gene‐test could provide farmers and veterinarians with information regarding management and treatment of herds.

## Ethics Statement

Animal owners agreed to the participation of their animals in this study; blood samples were collected based on routine procedures on these farm animals.

## Conflicts of Interest

The authors declare no conflicts of interest.

## Permission to Reproduce Material From Other Sources

Not applicable.

## Supporting information


Table S1.

## Data Availability

Genotypes of 10 SNPs and case and control information for the four herds are added as supplementary Table S1.

## References

[jbg12904-bib-0001] Abdel‐Shafy, H. , R. H. Bortfeldt , M. Reissmann , and G. A. Brockmann . 2014. “Short Communication: Validation of Somatic Cell Score—Associated Loci Identified in a Genome‐Wide Association Study in German Holstein Cattle.” Journal of Dairy Science 97: 2481–2486. 10.3168/jds.2013-7149. 24485673

[jbg12904-bib-0002] Abdel‐Shafy, H. , R. H. Bortfeldt , M. Reissmann , and G. A. Brockmann . 2018. “Short Communication: Validating Genome‐Wide Associated Signals for Clinical Mastitis in German Holstein Cattle.” Animal Genetics 49: 82–85. 10.1111/age.12624. 29314139

[jbg12904-bib-0003] Abdel‐Shafy, H. , R. H. Bortfeldt , J. Tetens , and G. A. Brockmann . 2014. “Single Nucleotide Polymorphism and Haplotype Effects Associated With Somatic Cell Score in German Holstein Cattle.” Genetics Selection Evolution 46: 35. 10.1186/1297-9686-46-35. PMC4078941 24898131

[jbg12904-bib-0004] Abebe, R. , H. Hatiya , M. Abera , B. Megersa , and K. Asmare . 2016. “Bovine Mastitis: Prevalence, Risk Factors and Isolation of *Staphylococcus aureus* in Dairy Herds at Hawassa Milk Shed, South Ethiopia.” BMC Veterinary Research 12: 270. 10.1186/s12917-016-0905-3. 27912754 PMC5135792

[jbg12904-bib-0005] Bianchi, R. M. , C. I. Schwertz , B. S. de Cecco , et al. 2019. “Pathological and Microbiological Characterization of Mastitis in Dairy Cows.” Tropical Animal Health and Production 51: 2057–2066. 10.1007/s11250-019-01907-0. 31073889

[jbg12904-bib-0006] Cobirka, M. , V. Tancin , and P. Slama . 2020. “Epidemiology and Classification of Mastitis.” Animals 10: 2212. 10.3390/ani10122212. 33255907 PMC7760962

[jbg12904-bib-0007] de Haas, Y. , H. W. Barkema , and R. F. Veerkamp . 2002. “The Effect of Pathogen‐Specific Clinical Mastitis on the Lactation Curve for Somatic Cell Count.” Journal of Dairy Science 85: 1314–1323. 10.3168/jds.S0022-0302(02)74196-9. 12086069

[jbg12904-bib-0008] De Vliegher, S. , I. Ohnstad , and S. Piepers . 2018. “Management and Prevention of Mastitis: A Multifactorial Approach With a Focus on Milking, Bedding and Data‐Management.” Journal of Integrative Agriculture 17: 1214–1233. 10.1016/S2095-3119(17)61893-8.

[jbg12904-bib-0009] Do, D. N. , F. S. Schenkel , F. Miglior , X. Zhao , and E. M. Ibeagha‐Awemu . 2018. “Genome Wide Association Study Identifies Novel Potential Candidate Genes for Bovine Milk Cholesterol Content.” Scientific Reports 8: 13239. 10.1038/s41598-018-31427-0. 30185830 PMC6125589

[jbg12904-bib-0010] Duse, A. , K. Persson‐Waller , and K. Pedersen . 2021. “Microbial Aetiology, Antibiotic Susceptibility and Pathogen‐Specific Risk Factors for Udder Pathogens From Clinical Mastitis in Dairy Cows.” Animals 11: 2113. 10.3390/ani11072113. 34359241 PMC8300163

[jbg12904-bib-0011] Elzaki, S. , P. Korkuc , D. Arends , M. Reissmann , S. A. Rahmatalla , and G. A. Brockmann . 2022. “Validation of Somatic Cell Score‐Associated SNPs From Holstein Cattle in Sudanese Butana and Butana × Holstein Crossbred Cattle.” Tropical Animal Health and Production 54: 50. 10.1007/s11250-022-03048-3. 35022894 PMC8755676

[jbg12904-bib-0012] Foerster, E. G. , T. Mukherjee , L. Cabral‐Fernandes , J. D. B. Rocha , S. E. Girardin , and D. J. Philpott . 2022. “How Autophagy Controls the Intestinal Epithelial Barrier.” Autophagy 18: 86–103. 10.1080/15548627.2021.1909406. 33906557 PMC8865220

[jbg12904-bib-0014] Hagnestam‐Nielsen, C. , U. Emanuelson , B. Berglund , and E. Strandberg . 2009. “Relationship Between Somatic Cell Count and Milk Yield in Different Stages of Lactation.” Journal of Dairy Science 92: 3124–3133. 10.3168/jds.2008-1719. 19528590

[jbg12904-bib-0015] Heikkilä, A. M. , E. Liski , S. Pyörälä , and S. Taponen . 2018. “Pathogen‐Specific Production Losses in Bovine Mastitis.” Journal of Dairy Science 101: 9493–9504. 10.3168/jds.2018-14824. 30122416

[jbg12904-bib-0016] Kreuzer, S. , M. Reissmann , and G. A. Brockmann . 2013. “Gene Test to Elucidate the ETEC F4ab/F4ac Receptor Status in Pigs.” Veterinary Microbiology 162: 293–295. 10.1016/j.vetmic.2012.07.049. 22917838

[jbg12904-bib-0017] Krömker, V. , and S. Leimbach . 2017. “Mastitis Treatment‐Reduction in Antibiotic Usage in Dairy Cows.” Reproduction in Domestic Animals 52, no. Suppl 3: 21–29. 10.1111/rda.13032. 28815847

[jbg12904-bib-0018] Lei, H. 2019. Impact of Genetics on Meat Quality of Pigs and Beef Cattle, Agricultural, Food and Nutritional Science, 275. University of Alberta.

[jbg12904-bib-0020] Macian, F. 2005. “NFAT Proteins: Key Regulators of T‐Cell Development and Function.” Nature Reviews. Immunology 5: 472–484. 10.1038/nri1632. 15928679

[jbg12904-bib-0021] Manning, S. E. , H. C. Ku , D. F. Dluzen , C. Xing , and Z. Zhou . 2023. “A Nonparametric Alternative to the Cochran‐Armitage Trend Test in Genetic Case‐Control Association Studies: The Jonckheere‐Terpstra Trend Test.” PLoS One 18: e0280809. 10.1371/journal.pone.0280809. 36730335 PMC9894441

[jbg12904-bib-0022] Narayana, S. G. , E. de Jong , F. S. Schenkel , et al. 2023. “Underlying Genetic Architecture of Resistance to Mastitis in Dairy Cattle: A Systematic Review and Gene Prioritization Analysis of Genome‐Wide Association Studies.” Journal of Dairy Science 106: 323–351. 10.3168/jds.2022-21923. 36333139

[jbg12904-bib-0023] Olde Riekerink, R. G. , H. W. Barkema , D. F. Kelton , and D. T. Scholl . 2008. “Incidence Rate of Clinical Mastitis on Canadian Dairy Farms.” Journal of Dairy Science 91: 1366–1377. 10.3168/jds.2007-0757. 18349229

[jbg12904-bib-0024] Pyörälä, S. 2008. “Mastitis in Post‐Partum Dairy Cows.” Reproduction in Domestic Animals 43, no. s2: 252–259. 10.1111/j.1439-0531.2008.01170.x. 18638132

[jbg12904-bib-0025] Rainard, P. , F. B. Gilbert , P. Germon , and G. Foucras . 2021. “Invited Review: A Critical Appraisal of Mastitis Vaccines for Dairy Cows.” Journal of Dairy Science 104: 10427–10448. 10.3168/jds.2021-20434. 34218921

[jbg12904-bib-0026] Shaheen, T. , S. Bilal Ahmad , M. U. Rehman , et al. 2020. “Investigations on Cytokines and Proteins in Lactating Cows With and Without Naturally Occurring Mastitis.” Journal of King Saud University, Science 32: 2863–2867. 10.1016/j.jksus.2020.07.009.

[jbg12904-bib-0027] Statello, L. , C. J. Guo , L. L. Chen , and M. Huarte . 2021. “Gene Regulation by Long Non‐Coding RNAs and Its Biological Functions.” Nature Reviews Molecular Cell Biology 22: 96–118. 10.1038/s41580-020-00315-9. 33353982 PMC7754182

[jbg12904-bib-0028] Stein, R. A. , and D. E. Katz . 2017. “Escherichia Coli, Cattle and the Propagation of Disease.” FEMS Microbiology Letters 364: fnx050. 10.1093/femsle/fnx050. 28333229 PMC7108533

[jbg12904-bib-0029] Valde, J. P. , M. L. Lystad , E. Simensen , and O. Østerås . 2007. “Comparison of Feeding Management and Body Condition of Dairy Cows in Herds With Low and High Mastitis Rates.” Journal of Dairy Science 90: 4317–4324. 10.3168/jds.2007-0129. 17699052

[jbg12904-bib-0030] Xu, C. , W. Zhang , Y. Jiang , et al. 2023. “Genome‐Wide Detection and Analysis of Copy Number Variation in Anhui Indigenous and Western Commercial Pig Breeds Using Porcine 80K SNP BeadChip.” Genes 14, no. 3: 654. 10.3390/genes14030654. 36980927 PMC10047991

[jbg12904-bib-0031] Yang, C. , S. W. Tsaih , A. Lemke , M. J. Flister , M. S. Thakar , and S. Malarkannan . 2018. “mTORC1 and mTORC2 Differentially Promote Natural Killer Cell Development.” eLife 7: e35619. 10.7554/eLife.35619. 29809146 PMC5976438

